# Measuring tissue sodium concentration: Cross‐vendor repeatability and reproducibility of ^23^Na‐MRI across two sites

**DOI:** 10.1002/jmri.26705

**Published:** 2019-03-12

**Authors:** Frank Riemer, Damien McHugh, Fulvio Zaccagna, Daniel Lewis, Mary A. McLean, Martin J. Graves, Fiona J. Gilbert, Geoff J.M. Parker, Ferdia A. Gallagher

**Affiliations:** ^1^ Department of Radiology University of Cambridge Cambridge UK; ^2^ CRUK & EPSRC Cancer Imaging Centre in Cambridge & Manchester UK; ^3^ Division of Neuroscience & Experimental Psychology The University of Manchester Manchester UK; ^4^ Cancer Research UK Cambridge Institute University of Cambridge Cambridge UK; ^5^ Bioxydyn Ltd. Manchester UK

**Keywords:** sodium MRI, brain, repeatability, reproducibility, biomarker

## Abstract

**Background:**

Sodium MRI (^23^Na‐MRI)‐derived biomarkers such as total sodium concentration (TSC) have the potential to provide information on tumor cellularity and the changes in tumor microstructure that occur following therapy.

**Purpose:**

To evaluate the repeatability and reproducibility of TSC measurements in the brains of healthy volunteers, providing evidence for the technical validation of ^23^Na‐MRI‐derived biomarkers.

**Study Type:**

Prospective multicenter study.

**Subjects:**

Eleven volunteers (32 ± 6 years; eight males, three females) were scanned twice at each of two sites.

**Field Strength/Sequence:**

Comparable 3D‐cones ^23^Na‐MRI ultrashort echo time acquisitions at 3T.

**Assessment:**

TSC values, quantified from calibration phantoms placed in the field of view, were obtained from white matter (WM), gray matter (GM), and cerebrospinal fluid (CSF), based on automated segmentation of coregistered ^1^H T_1_‐weighted images and hand‐drawn regions of interest by two readers.

**Statistical Tests:**

Coefficients of variation (CoVs) from mean TSC values were used to assess intrasite repeatability and intersite reproducibility.

**Results:**

Mean GM TSC concentrations (52.1 ± 7.1 mM) were ∼20% higher than for WM (41.8 ± 6.7 mM). Measurements were highly repeatable at both sites with mean scan–rescan CoVs between volunteers and regions of 2% and 4%, respectively. Mean intersite reproducibility CoVs were 3%, 3%, and 6% for WM, GM, and CSF, respectively.

**Data Conclusion:**

These results demonstrate technical validation of sodium MRI‐derived biomarkers in healthy volunteers. We also show that comparable ^23^Na imaging of the brain can be implemented across different sites and scanners with excellent repeatability and reproducibility.

**Level of Evidence**: 1

**Technical Efficacy**: Stage 2

J. Magn. Reson. Imaging 2019;50:1278–1284.

QUANTIFYING AND IMAGING sodium distribution provides an important biomarker of normal tissue function and of the changes that can occur during different disease processes due to the importance of sodium ions in many aspects of cell physiology. Viable cells maintain a sodium gradient across the cell membrane with a high extracellular sodium concentration (140–150 mM) and a low intracellular sodium concentration (10–15 mM).^1^ This transmembrane gradient is generated and maintained through the action of the Na+/K+ ATPase (sodium‐potassium pump) that exports three Na+ ions out of the cell in exchange for two K+ ions, and this ionic gradient is then used to drive several other membrane‐bound exchangers and cotransporters.^2^ The sodium‐23 (^23^Na) nucleus gives the second strongest nuclear magnetic resonance (NMR) signal from biological tissues after the water proton (^1^H).^3^ However, despite the overall sensitivity of ^23^Na being 10 times lower than that of ^1^H, sodium distribution has been successfully imaged in vivo using magnetic resonance imaging (MRI).

The lower concentration of ^23^Na relative to water protons and its short biexponential T_2_ relaxation time are significant challenges for the implementation of sodium MRI.^4^ However, advances in MRI hardware and tailored ^23^Na MRI sequences^5^ have facilitated higher resolution clinical ^23^Na MRI as a method to probe tissue biology in health and disease. There is ongoing research into the use of ^23^Na MRI in many conditions ranging from musculoskeletal and neurodegenerative diseases to stroke.^3^, ^6^, ^7^

Several studies have shown intracellular sodium volume to be significantly higher in malignant tumors compared with benign lesions due to increased cell density, altered metabolism, and changes in cell membrane ion transporters.^8^, ^9^ In addition to this increase in intracellular sodium, many malignant tumors also show an increase in the sodium‐rich extravascular–extracellular space, which in some cases may be secondary to an increase in necrosis.^10^, ^11^, ^12^, ^13^, ^14^ Combining information on sodium concentration from both the intracellular and the extracellular compartments provides complementary information that can be used to probe tissue structure, cell density, and membrane integrity in more detail, which could be used in the assessment of tumor progression and treatment response.^15^ ^23^Na MRI has been shown to be sensitive to therapy‐induced changes in both breast and central nervous system tumors.^16^ In a recent study, ^23^Na MRI outperformed an invasive histological correlate for brain tumor prognostication.^17^ ^23^Na MRI may therefore allow better assessment of tissue cellularity and may allow treatment to be more personalized as a result of documenting the variety of responses seen within and between tumors.

The most widely reported metric in ^23^Na MRI studies is the total sodium concentration (TSC), which is the combination of both the intracellular and extracellular contributions. Before such biomarkers can be clinically utilized, they require both technical and biological validation to establish their accuracy and precision, as well as their relationship to cancer biology. Repeatability and reproducibility need to be demonstrated at an early stage of imaging biomarker development. While a number^4^, ^18^, ^19^, ^20^, ^21^ of studies report low standard deviations in normal tissue sodium concentrations, absolute TSC results and acquisition methods vary substantially between different studies and different centers, making comparisons difficult. As such, the purpose of this work was to implement comparable ^23^Na MRI acquisition protocols at different sites, and evaluate the repeatability (intrasite variation) and reproducibility (intersite variation) of TSC measurements, providing data contributing to the technical validation of ^23^Na MRI‐derived biomarkers.

## Materials and Methods

### *Participants*

Local research Ethics Committee approval was granted, and volunteers provided informed consent. The brains of 11 healthy volunteers (32 ± 6 years; eight males, three females), with no history of neurological disease, were scanned at two sites (A and B) with different MRI scanners. At site A, all the volunteers were scanned twice; at site B, seven were scanned twice and four once. The two scans at a given site were <1 hour apart and were used to assess repeatability (intrasite variation); scans for a given volunteer at both sites ranged from 1–41 days apart and were used to assess reproducibility (intersite variation).

### *Calibrants*

Two batches of sodium calibration phantoms were made from a stock of 20 mM and 80 mM sodium chloride (NaCl) in water with 4% agar and 1% nickel sulfate (NiSO_4_) and distributed to the two sites. Each of the phantoms were cylindrical tubes of 30 ml capacity (physical dimensions 2.45 × 8.9 cm; Dutscher, France), filled with the stock solution. These were placed beside the ear defenders/headphones worn by the subjects to include them in the imaging field of view (FOV). An in‐house script written in MatLab v. 9.1 (MathWorks, Natick, MA) was used to automatically outline the phantoms and erode the voxels at the periphery, which are most likely to suffer from partial volume based on intensity thresholding.

### *MRI Acquisition*

The sodium acquisition was performed using a matched protocol 3D‐Cones UTE sequence. The 3D‐Cones trajectory is attractive for this type of study: the code for waveform generation is freely available; the trajectory is utilizable with both 3D and UTE imaging, therefore increasing signal‐to‐noise ratio (SNR) over standard 2D gradient echo sequences; and it has a high sampling efficiency, allowing for shorter scans compared with 3D radial UTE sequences.^22^ The two 3 T systems were from different MR manufacturers (GE MR750, GE Healthcare, Waukesha, WI; and Achieva, Philips Medical Systems, Best, The Netherlands). Both systems used the same model of ^1^H/^23^Na dual‐tuned head coil (RAPID Biomedical, Rimpar, Germany). The 3D‐Cones trajectory was computed offline and imported into the respective scanner environments, making the gradient waveforms and the main sequence parameters identical on each system: 4 mm isotropic nominal resolution, 24 × 24 cm FOV, 10 msec readout, 2184 readouts, 3 averages, 166 kHz full readout bandwidth, 30 mT/m maximum gradient amplitude, 120 mT/s maximum slew rate. The echo time (TE) and repetition time (TR) were 0.5 msec and 100 msec for both systems.

To account for gradient timing errors, phantom imaging at both sites was undertaken to optimize the line profile through a homogenous phantom. The difference between actual and theoretical waveform was corrected by convolving the reconstruction trajectory with an exponential hysteresis function that shifts the *k*‐space samples in time. An experimentally determined delay of 5 μs provided the best line profile (straight through the phantom, abrupt at phantom edges) for both sites.

A volumetric T_1_‐weighted (T_1_W) fast spoiled gradient echo (FSPGR) sequence was acquired at both sites with 1 mm isotropic resolution, 24 × 24 cm FOV, TE/TR = 2.6/7 msec, 62.5 kHz full readout bandwidth for segmentation and region of interest (ROI) analysis.

### *Data Analysis*

Images were reconstructed in MatLab v. 9.1.^23^ A sample density weighted apodization^24^ was used to match the SNR between the sites. TSC maps were generated based on the calibration phantoms placed in the FOV.^25^ TSC values were obtained from white matter (WM), gray matter (GM), and cerebrospinal fluid (CSF), based on automated segmentation of coregistered ^1^H T_1_W images (SPM12, UCL, London, UK). To reduce the effect of partial volume, only voxels with a tissue probability larger than 95% were included in the masks. In addition, a hand‐drawn ROI analysis was performed using Osirix 8.5 (Pixmeo SARL, Switzerland) by two observers (6 and 12 years of experience with radiological imaging) to assess interobserver agreement.

The selection of the anatomical structures included in the analysis were performed by two observers in consensus to reduce bias. The caudate and the putamen were selected as representative regions of deep GM; the centrum semiovale was chosen to represent WM. Care was taken to diminish the potential partial volume effect of the ventricles on the ROIs and all the ROIs were drawn bilaterally. Coefficients of variation (CoVs) of mean values were used to assess repeatability (between scans 1 and 2 at a given site) and reproducibility (between the first scan at each site).

## Results

The phantoms appeared on imaging with a diameter of at least 6 voxels over 20 slices and each individual 2D phantom ROI that was obtained contained 10–30 voxels.

**Figure 1:**
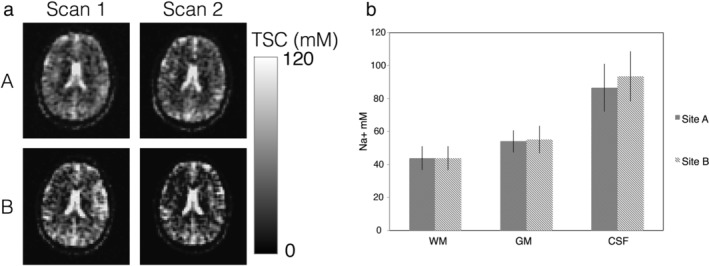
Mean total sodium concentrations across brain regions from the two sites: **(a)** Example TSC maps from one subject scanned twice at sites A and B. **(b)** Mean TSC ± SD for the first scan and all volunteers in segmented WM, GM, and CSF for sites A and B.

Example TSC maps from one subject scanned twice at both sites are shown in Fig. 1a, with WM, GM, and CSF values averaged over the first scan from all subjects using the segmentation masks is shown in Fig. 1b. Qualitatively, TSC values in segmented regions were consistent across sites, with mean GM concentrations slightly higher than WM, and CSF values generally higher than WM and GM. Minor qualitative differences between sites A and B are shown in Fig. 1a.

**Figure 2:**
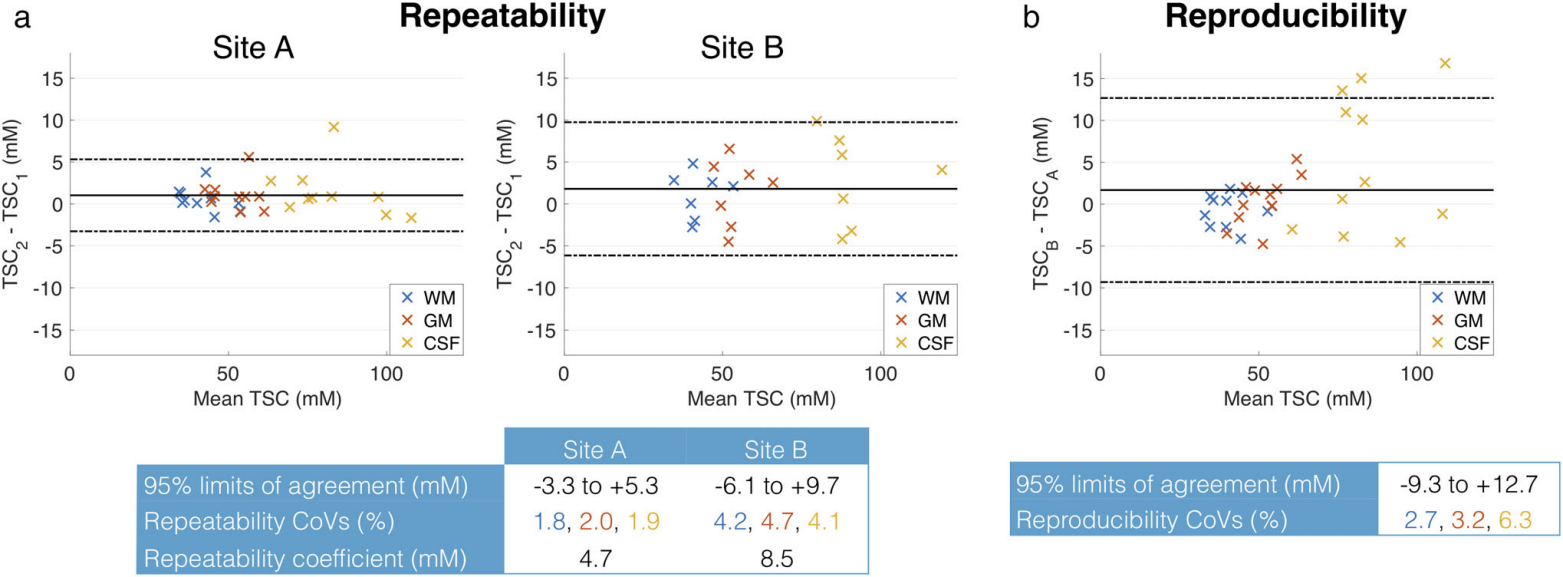
Bland–Altman plots of repeatability and reproducibility for automated segmentation. **(a)** Repeatability of TSC in segmented brain regions, comparing scans 1 and 2 at each site. Individual data points represent the difference in mean TSC between scans for a given subject, plotted against the mean of the two TSC measurements. **(b)** Reproducibility of TSC in segmented brain regions, comparing the first scan at each site.

The repeatability and reproducibility of segmented TSC values are presented with Bland–Altman plots in Fig. 2a,b, respectively. Measurements were more repeatable at site A than B, with mean repeatability coefficients of 4.7 mM and 8.5 mM, respectively. The limits of agreement at the 95% confidence interval (CI) were –3.3 to +5.3 mM and –6.1 to +9.7 mM at sites A and B, respectively. The repeatability CoVs for WM, GM, and CSF were 1.8, 2.0, and 1.9% for site A and 4.2, 4.7, and 4.1%, respectively, for site B. The reproducibility CoVs between site A and B were 2.7, 3.2, and 6.3% for WM, GM, and CSF. At the 95% CI, the limits of agreement of reproducibility between site A and B were –9.3 to +12.7 mM with greater variation in CSF (Fig. 2b). Mean sodium concentration estimates for the segmentation analysis for each scan at the two sites are given in Table 1.

**Table 1:** Mean Total Sodium Concentrations (mM) From the Segmentation Analysis for All Subjects Given for Each Site and Each Scan

	Site A			Site B				
	Scan 1	Scan 2	Mean	Scan 1	Scan 2	Mean	Site A & B	Mean
*WM*	41.6 ± 7.3	42.4 ± 7.1	42.0 ± 6.9	41.1 ± 7.3	43.0 ± 6.3	41.8 ± 6.7	*WM*	41.8 ± 6.7
*GM*	51.0 ± 6.5	52.3 ± 6.7	51.8 ± 6.5	51.6 ± 8.6	54.6 ± 6.9	52.7 ± 7.8	*GM*	52.1 ± 7.1
*CSF*	81.8 ± 14.3	83.7 ± 13.9	82.7 ± 13.8	86.9 ± 15.7	93.0 ± 12.8	89.2 ± 14.5	*CSF*	85.8 ± 14.3

**Figure 3:**
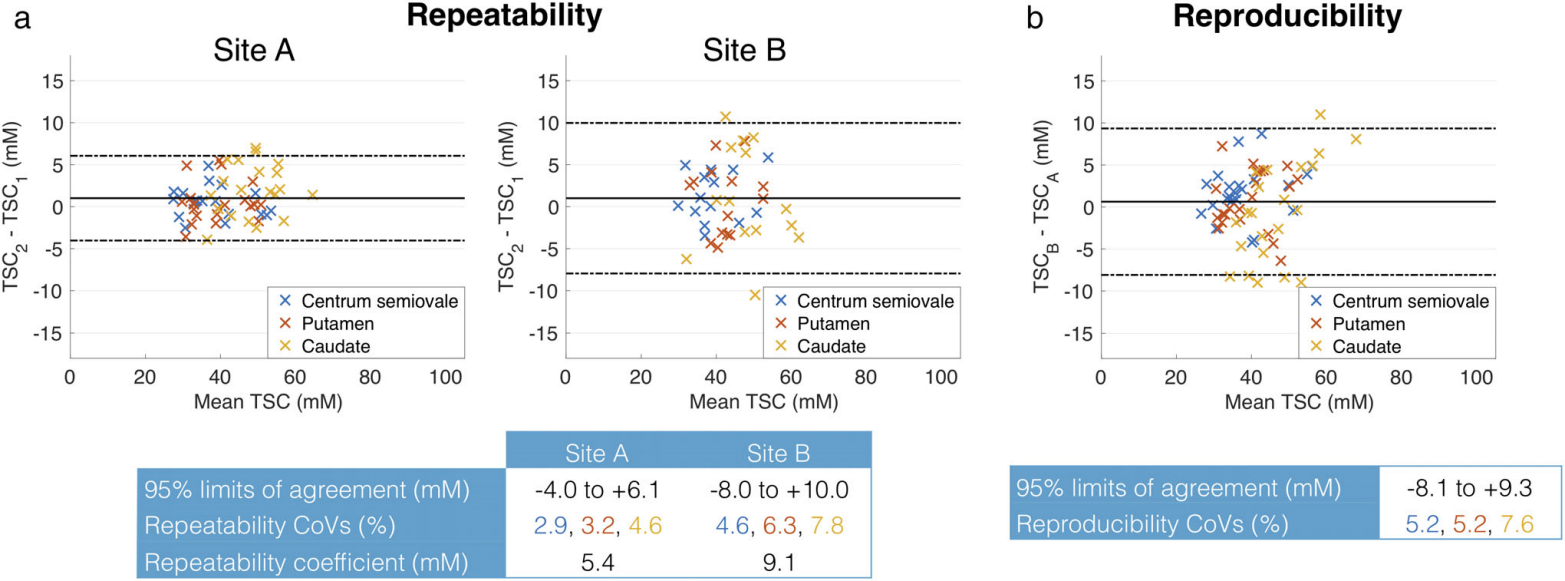
Bland–Altman plots of repeatability and reproducibility for manual segmentation. **(a)** Repeatability of TSC in three manually defined ROIs, comparing scans 1 and 2 at each site. Individual data points represent the difference in mean TSC between scans for a given subject, plotted against the mean of the mean TSCs. **(b)** Reproducibility of TSC in manually defined ROIs, comparing subjects' first scan at each site.

The repeatability and reproducibility of TSC in hand‐drawn ROIs are shown in Fig. 3a,b, respectively, for one observer. As with the segmentation analysis, repeatability was better at site A than B, with repeatability coefficients of 5.4 mM and 9.1 mM, respectively (Fig. 3a). The limits of agreement at the 95% CI were –4.0 to +6.1 mM and –8.0 to +10.0 mM at sites A and B, respectively. The repeatability CoVs for centrum semiovale, putamen, and caudate were 2.9, 3.2, and 4.6% for site A and 4.6, 6.3, and 7.8%, respectively, for site B. The reproducibility CoVs between site A and B were 5.2, 5.2, and 7.6% for centrum semiovale, putamen, and caudate. At the 95% CI, interval, the limits of agreement of reproducibility between site A and B were –8.1 to +9.3 mM (Fig. 3b).

**Figure 4:**
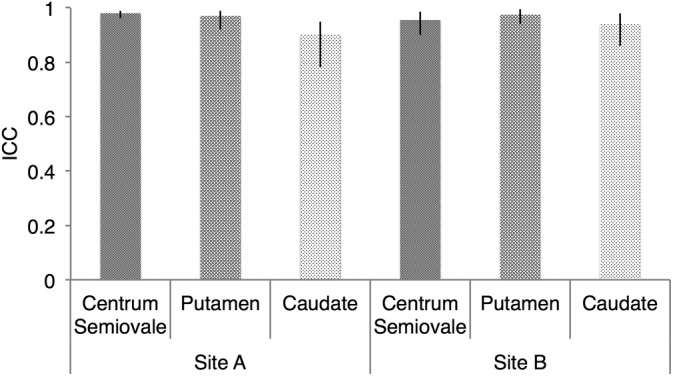
Assessment of interobserver agreement using ICCS. Data points and error bars represent the ICC and 95% CI for TSC values in ROIs drawn by the two observers. ICCs were calculated separately for each ROI for the first scan at sites A and B.

The results of the hand‐drawn ROI analysis described above come from one observer. Similar results were obtained from the second observer, with intraclass correlation coefficients (ICCs) used to formally assess interobserver agreement. ICCs calculated separately for each ROI in the first scan at each site generally indicated good interobserver agreement (Fig. 4), with ICC point estimates >0.85 in all cases. Mean sodium concentration estimates for the ROI analysis for each observer are given in Table 2.

**Table 2:** Mean Sodium Concentrations (mM) From the ROI Analysis for All Subjects at Each Site and for Each Observer

	Site A			Site B			
	Observer 1	Observer 2	Mean	Observer 1	Observer 2	Mean	Site A & B Mean
*Caudate*	48.1 ± 7.1	47.8 ± 6.3	47.9 ± 6.7	47.2 ± 8.0	46.6 ± 7.5	47.0 ± 7.6	47.6 ± 7.1
*Centrum Semiovale*	37.6 ± 8.0	37.2 ± 8.0	37.4 ± 8.0	39.5 ± 8.2	39.9 ± 8.4	39.6 ± 11.3	38.6 ± 8.0
*Putamen*	39.3 ± 6.3	39.9 ± 7.1	39.5 ± 7.1	41.1 ± 6.9	42.4 ± 7.0	41.6 ± 6.9	40.5 ± 7.1

## Discussion

This study assessed the repeatability and reproducibility of total brain tissue sodium as well as the first to utilize scanners from different vendors. Furthermore, it is the first to compare the use of a 3D Cones acquisition that has been harmonized across platforms.

The total sodium concentration measurements acquired in this study from automated segmentation of white and gray matter analysis are consistent with previous studies,^19^, ^21^, ^26^ eg, 41.8 ± 6.9 vs. 45.9 ± 3.1 mM, respectively, for WM and 52.1 ± 7.1 vs. 52.7 ± 3.6 mM for GM.^26^ WM measurements from the hand‐drawn ROI analysis here are also in agreement with previous studies: for example, Qian et al^27^ showed 37.4 ± 2.9 vs. 38.6 ± 8.0 mM here. GM values also showed good agreement with the literature: 47.6 ± 7.1 for the caudate here vs. 52.0 ± 6.0 mM^28^; and 40.9 ± 7.1 for the putamen here vs. 43.0 ± 3.0 mM.^28^ In general, differences between overall GM and the putamen are unsurprising due to cellular differences between deep GM and cortical GM: for example, the basal ganglia is permeated by a small amount of WM fiber bundles which would give a TSC that is a composite between WM and GM. Cortical GM, however, is more likely to be affected by partial volume effects due to the proximity of the CSF. The caudate GM values also demonstrate lower reproducibility, possibly due to partial volume effects, as it is bordering on WM and CSF.

Within our study cohort, measured CSF concentration values demonstrated poorer reproducibility compared with WM and GM measurements, which may be due to partial volume effects from surrounding tissue, real differences in sodium CSF concentration, or potential signal loss due to flow and pulsation.^29^ The CSF spaces are typically small in this young healthy cohort (average age 32 ± 6 years), leading to a higher risk of partial volume that may impact the absolute concentration measurement. In healthy volunteers, CSF flows at a rate of 11.1 ± 4.9 ml/min and changes in Na^+^ ion concentration have been reported in relation to migraine onset,^30^ suggesting that the CSF may be more subject to short‐term changes in sodium concentration.

Absolute sodium ion concentration within the CSF from studies that use nonimaging methods has been reported in the range of 135–155 mM,^30^ but exact values are rarely reported in ^23^Na MRI studies due to the partial T_1_‐recovery saturation issues at short repetition time (the T_1_ of CSF is ∼47 msec, compared with the T_1_ of brain tissue, which is ∼22 msec).^31^ Nevertheless, in a study undertaken by Ouwerkerk et al,^32^ the mean sodium concentration of CSF, derived using ^23^Na MRI at 1 .5T in a cohort of healthy volunteers (age range 22–63 years), was 135 ± 15 mM. While this value is higher than that reported in our study, the standard deviation in CSF values was large and is not inconsistent with the range of CSF measurement values (69.2–121.3) seen across our patient cohort. Furthermore, while Ouwerkerk et al's^32^ result was calculated from a hand‐drawn ROI placed within the lateral ventricles, a segmentation mask that included contributions from all CSF containing regions was used in our study. Due to the lack of appreciable brain atrophy within the young, healthy cohort studied, the cortical CSF spaces and lateral ventricles are often small, increasing the risk for partial volume effects, and potentially thereby lowering measured CSF concentration values within the subarachnoid and ventricular space respectively.

Qualitative differences in the appearance of the images between sites A and B such as blurring and contrast between brain parenchyma and CSF could be due to variations in eddy current behavior. Blurring of images related to eddy currents in spiral‐like imaging has previously been shown.^33^ Blurring may increase the risk of partial volume due to a poorer point spread function; however, in this study site A did not perform less well than site B in reproducibility and repeatability.

The small CoVs of our results confirm that TSC is highly reproducible between sites and observers. Madelin et al^35^ assessed repeatability of fluid‐suppressed sodium concentration in the brain by scanning 11 subjects twice on the same system using the FLORET sequence^34^ and found CoVs for estimated parameters between 10–20%.^35^ The higher CoVs in that study may be due to the lower SNR nature of fluid suppressed imaging and the advent of ^23^Na imaging as compared with the total sodium imaging utilized in our study. In a different study, Madelin et al^36^ looked at reproducibility and repeatability of total sodium imaging and fluid suppressed sodium concentration in the articular cartilage of six volunteers utilizing two different systems (3 T and 7 T, same vendor) and found CoVs over both systems to be between 7.5–13.6% using a radial sequence.^36^ Newbould et al and Jordan et al^37^, ^38^ both looked at repeatability of total sodium MRI measures in articular cartilage by scanning the same subjects using a 3D Cones sequence and found CoVs of 3.2% and 11.3%, respectively. Given the small size of articular cartilage and the involved potential partial volume issues compared with the comparatively larger brain structures investigated in this study, the smaller CoVs observed in our study are to be expected.

Previous authors have demonstrated increased TSC within CNS neoplasms such as high‐grade glioma,^17^, ^32^, ^39^ and that ^23^Na MRI can be used as a predictive marker of both tumor grade^32^ and prognosis.^17^ Within glioma, cellular proliferation and changes in cell membrane ion transporters^17^ contribute to elevated intracellular sodium concentrations, but these tumors also display increases in the sodium‐rich extravascular–extracellular space secondary to necrosis and loss of normal cellular packing.^10^, ^11^, ^12^, ^13^, ^14^ Evidence from in vivo animal studies of temporal ^23^Na MRI changes in glioma following chemotherapy treatment suggest that TSC may also serve as an early marker of progression and treatment response in this tumor group.^40^ While nonresponsive tumors in this study displayed a progressive increase in measured TSC, within responsive glioma regions there was an early sharp rise in TSC, an effect they hypothesized was due to therapy‐induced loss of membrane integrity and later cellular necrosis.^40^ Further studies should be undertaken to assess treatment and tumor growth‐related TSC changes in human glioma, but our study findings that these measurements demonstrate good repeatability and reproducibility in normal brain represent an early important step in the development of TSC as an imaging biomarker in CNS tumors.

The present study has a number of limitations, including technical aspects and study design. The true resolution of non‐Cartesian imaging studies is also difficult to define due to the nonuniform sampling and blurring introduced by dephasing of short T_2_ species during readout. The real resolution obtained is likely to be larger than the nominal resolution. However, by adapting the readout length to be shorter than the average expected sodium T_2_s, the effect of blurring was minimized in our study. Modeling of the image point‐spread‐function and correction in postprocessing was not used in our study and may improve estimates in future studies. B_0_ ± B_1_ correction are not a major concern at our field strength due to the low gyromagnetic ratio of sodium and the use of a volume resonator. Studies investigating ^23^Na at higher field strengths and/or using phased arrays will benefit from such corrections. In terms of technical limitations, an important consideration is the potential influence of relaxation on TSC measurements. Although a short TE was used to reduce T_2_* weighting, changes in transverse relaxation and/or tissue compartments may confound TSC measurements. Similarly, while the chosen TR matches that used in previous studies and provides a reasonable compromise between SNR, scan time, and T_1_‐weighting, TSC comparisons between tissues with different and long (>20 msec) T_1_ may be confounded. These limitations may be addressed in future studies by incorporating ^23^Na relaxation time measurements. In terms of study design, limitations include the small number of subjects, and their reasonably narrow age range. While including more subjects over a broader age range would be beneficial, we do not think the conclusions would be substantially altered, especially given the evidence that TSC shows little variation in cognitively normal aging.^43^ The restriction of recruiting only healthy volunteers is another limitation, with the present study providing results against which pathology‐specific repeatability measurements can be compared. Finally, comparisons among more sites and scanners is crucial if ^23^Na MRI is to yield robust biomarkers.

In conclusion, this study demonstrated good intersite and intrasite reproducibility and repeatability for ^23^Na MRI of the normal human brain. This work can be used to inform biologically meaningful differences in sodium concentration that could be used in the future as a biomarker in multisite studies. This is a key step in establishing sodium MRI‐derived metrics of tissue microstructure in cancer and other diseases.

## Conflict of Interest

The authors declare no conflicts of interest.

## Data Availability

All data are available on reasonable request from the authors.
